# Kinetic Studies on the Reaction between Dicyanocobinamide and Hypochlorous Acid

**DOI:** 10.1371/journal.pone.0110595

**Published:** 2014-11-06

**Authors:** Dhiman Maitra, Iyad Ali, Rasha M. Abdulridha, Faten Shaeib, Sana N. Khan, Ghassan M. Saed, Subramaniam Pennathur, Husam M. Abu-Soud

**Affiliations:** 1 Department of Obstetrics and Gynecology, The C.S. Mott Center for Human Growth and Development, Wayne State University School of Medicine, Detroit, MI, United States of America; 2 Department of Biochemistry and Genetics, Faculty of Medicine and Health Sciences, An-Najah National University, Nablus, Palestine; 3 Division of Nephrology, Department of Internal Medicine, University of Michigan Medical School, Ann Arbor, MI, United States of America; 4 Department of Biochemistry and Molecular Biology, The C.S. Mott Center for Human Growth and Development, Wayne State University School of Medicine, Detroit, MI, United States of America; Case Western Reserve University, United States of America

## Abstract

Hypochlorous acid (HOCl) is a potent oxidant generated by myeloperoxidase (MPO), which is an abundant enzyme used for defense against microbes. We examined the potential role of HOCl in corrin ring destruction and subsequent formation of cyanogen chloride (CNCl) from dicyanocobinamide ((CN)_2_-Cbi). Stopped-flow analysis revealed that the reaction consists of at least three observable steps, including at least two sequential transient intermediates prior to corrin ring destruction. The first two steps were attributed to sequential replacement of the two cyanide ligands with hypochlorite, while the third step was the destruction of the corrin ring. The formation of (OCl)(CN)-Cbi and its conversion to (OCl)_2_-Cbi was fitted to a first order rate equation with second order rate constants of 0.002 and 0.0002 µM^−1^s^−1^, respectively. The significantly lower rate of the second step compared to the first suggests that the replacement of the first cyanide molecule by hypochlorite causes an alteration in the ligand *trans* effects changing the affinity and/or accessibility of Co toward hypochlorite. Plots of the apparent rate constants as a function of HOCl concentration for all the three steps were linear with Y-intercepts close to zero, indicating that HOCl binds in an irreversible one-step mechanism. Collectively, these results illustrate functional differences in the corrin ring environments toward binding of diatomic ligands.

## Introduction

Cyanogen chloride (CNCl, CAS 506-77-4) is a disinfectant byproduct found in drinking water treated with free chlorine and chloramines at concentrations ranging from 0.45–0.8 µg/L [1], [2]. CNCl can be hydrolyzed to form hydrogen cyanide (HCN), and hypochlorous acid (HOCl) [3]. Cyanogen chloride and its reduced final product cyanide (CN^-^) are highly toxic, and their damage potential depends mainly on the concentration and duration of exposure [4]. Any exposure to higher concentrations causes immediate injury to multiple organs in the central nervous, cardiovascular, and pulmonary systems [5]. Prolonged exposure can cause permanent brain damage, muscle paralysis, coma, and death [4]. The ability of these molecules to react with sulfhydryl compounds such as protein thiols and reduced glutathione (GSH) causes the toxicity elicited in biological systems [6]. They are also known to block the electron transport chain by inhibiting mitochondrial cytochrome C oxidase, initiating a fatal series of events by decreased oxidative metabolism and oxygen utilization [7]. Recently, we have shown that significant amounts of CNCl/CN^-^ are liberated by mixing cyanocobalamin (Cbl), the most common supplemental form of vitamin B_12_, with HOCl through a mechanism that involves disruption of axial coordination of the Co atom and cleavage of the corrin ring [8].

**Figure 1:**
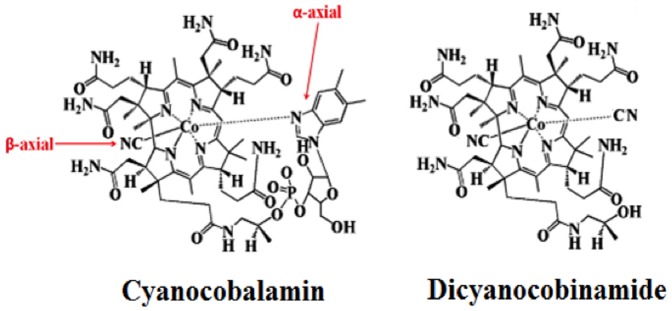
Structures of cyanocobalamin (Cbl) (left panel) and dicyanocobinamide ((CN)_2_-Cbi)) (right panel).

The structure of the Cbl and dicyanocobinamide ((CN)_2_-Cbi), a naturally occurring intermediate of vitamin B_12_ synthesis, are based on a corrin ring. In the corrin ring four of the six coordination sites of the cobalt (Co) atom are the pyrrole nitrogen atoms, which are provided by the corrin ring (Figure 1). In Cbl, the fifth position of the Co atom (the lower or α- axial ligand) is taken by one of the nitrogens of the 5,6- dimethylbenzimidazole group [9]. The other nitrogen of the 5,6-dimethylbenzimidazole is connected to a five-carbon sugar, which in turn links to a phosphate group, and then back to the corrin ring via one of the seven-amide groups at the periphery of the corrin ring. Finally, the sixth position (the upper/β-axial ligand, also called the site of reactivity, is occupied by a cyano group (-CN) (Figure 1A). Cobinamides (Cbi) are vitamin B_12_ derivatives that lack the dimethylbenzimidazole group at the α-axial ligand. The solubility, stability, and the CN^-^ binding ability of cobalamin derivatives depend on the type of the β-axial ligand and the presence or lack of 5,6-dimethylbenzimidazole at the α-axial ligand [10]–[16] The presence of this group in Cbi causes the increased binding ability to CN^-^ (100 times greater than Cbl), explaining the difference of Cbi as compared to Cbl in CN^-^ detoxification of massive CN^-^ poisoning [17], [18]. Dicyanocobinamide plays an important role, both in vitro and in intact cells, as a soluble guanylate cyclase (sGC) co-activator by targeting the N-terminal regulatory regions, an action which resembles the effect of forskolin on adenylyl cyclases [19]. It increases intracellular cGMP levels and displays vasorelaxant activity in phenylephrine-constricted rat aortic rings in an endothelium-independent manner. Both effects are synergistically potentiated by BAY41-2272, (an NO-independent soluble guanylate cyclase stimulator consisting of structurally diverse benzylindazole/pyrazolopyridine and acrylamide derivatives) [19]. Recently, it has been shown that (CN)_2_-Cbi and related vitamin B_12_ derivatives may serve not only as nitric oxide (NO) scavengers [20], but also as NO synthase (NOS) inhibitors [21]. They have also been shown to serve as potent inhibitors of HIV-1 integrase and may prove useful as anti-viral treatments [22].

Hypochlorous acide, which is produced by heme peroxidase and myeloperoxidase (MPO) using hydrogen peroxide (H_2_O_2_) and chloride (Cl^-^) as co-substrates, is a vital component of the innate immune system, which protects the host through the oxidative destruction of invading pathogens and microbes [23], [24]. Activated neutrophils have been previously found to generate around 150–425 µM of HOCl per hour [24]–[27], and it has been estimated that HOCl levels can reach up to 5 mM at sites of inflammation [28]. Furthermore, the progression of a number of pathological conditions including atherosclerosis, pulmonary fibrosis, vascular and diabetic complications, glomerulonephritis, cancer and even oocyte aging have been linked to high levels of reactive oxygen species (ROS) such as HOCl [29]–[33].

With the use of a combination of optical absorbance and rapid kinetic approaches, we now show that HOCl mediates (CN)_2_-Cbi corrin destruction through a complex multistep process. This sequence includes the generation of at least two kinetically and spectrophotometrically distinct intermediates prior to corrin ring destruction and the generation of CNCl. Collectively, these kinetic behaviors distinguish HOCl from other ROS, and provide a foundation to advance our understanding of the role of HOCl plays in destroying the tetrapyrrole-based compounds as well as the associated toxicity.

## Materials and Methods

### Materials

All the materials used were of highest purity grade and used without further purification. Sodium hypochlorite (NaOCl), pyridine, 1,3 dimethyl barbituric acid, Cbl, (CN)_2_-Cbi, L-methionine, methanol- HPLC grade, were obtained from Sigma Aldrich (St. Louis, MO, USA).

### Absorbance Measurements

A Cary 100 Bio UV–visible spectrophotometer was used to record the absorbance spectra, at 25°C, pH 7.0. Experiments were performed in a 1-ml phosphate buffer solution (200 mM, pH 7.4), and then supplemented with fixed amount of (CN)_2_-Cbi (10 µM) followed by increasing concentrations of HOCl. Reaction completion was assured after 2 hours of incubation, and methionine (5-fold of the final HOCl concentration) was added to eliminate excess HOCl, and absorbance changes were recorded from 300 to 700 nm.

### Rapid Kinetic Measurements

A dual syringe stopped-flow instrument from Hi-Tech, Ltd. (Model SF-61) was used to perform kinetic measurements of HOCl mediated (CN)_2_-Cbi destruction. Measurements were carried out in an aerobic atmosphere at 25°C and 37°C following rapid mixing of equal volumes of a buffer solution containing a fixed amount of (CN)_2_-Cbi (20 µM; initial), and a buffer solution containing increasing concentration of HOCl. The time course of the absorbance change was fitted to a single-exponential (Eq. 1), or a double-exponential (Eq. 2) function as indicated. Signal-to-noise ratios for all kinetic analyses were improved by averaging at least six to eight individual traces. In some experiments, the stopped-flow instrument was attached to a rapid scanning diode array device (Hi-Tech). This instrument was designed to collect multiple complete spectra (200–800 nm) for specific time ranges. The detector was automatically calibrated relative to a holmium oxide filter, as it had spectral peaks at 360.8, 418.5, 446.0, 453.4, 460.4, 536.4, and 637.5 nm, which were used by the software to correctly align pixel positions with wavelength.



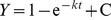
(Eq. 1)




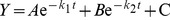
(Eq. 2)


### Colorimetric detection of CNCl

CNCl was detected colorimetrically using the pyridine-1,3 dimethyl barbituric acid reagent as previously reported [34]. The amount of CNCl was determined from the extinction coefficient of 1.03×10^5^ M^−1^cm^−1^ for the violet colored complex [35].

### pH Measurements

An experiment on the effect of pH on HOCl mediated (CN)_2_-Cbi corrin destruction was carried out using 50 mM sodium acetate buffer (pH 4.5 and 5.7), and 50 mM phosphate buffer (pH 6–9). The different spectra were collected at 25°C.

### Solution Preparation

#### HOCl preparation

HOCl was prepared using a slight modification of a previously published method [36]. Briefly, a stock solution of HOCl was prepared by adding 1 mL NaOCl solution to 40 mL of 154 mM NaCl and the pH was adjusted to approximately 3 by adding HCl. The concentration of active total chlorine species in solution expressed as [HOCl]_T_ (where [HOCl]_T_  =  [HOCl] + [Cl_2_] + [Cl_3_^−^] + [OCl^−^]) in 154 mM NaCl was determined by converting all the active chlorine species to OCl^−^ by adding a single bolus of 40 µL 5 M NaOH and measuring the concentration of OCl^−^. The concentration of OCl^−^ was determined spectrophotometrically at 292 nm (ε = 362 M^−1^ cm^−1^). Secondary to the fact that HOCl is unstable, the stock solution was freshly prepared daily, stored on ice, and used within one hour of preparation. For further experiments, dilutions were made from the stock solution using 200 mM phosphate buffer pH 7, to give working solutions of lower HOCl concentrations.

#### (CN)_2_-Cbi preparation

Dicyanocobinamide stock solution was prepared by dissolving an exact weight of (CN)_2_-Cbi in distilled water.

## Results

### Stability of (CN)_2_-Cbi and the distribution of chlorine species as a function of pH

**Figure 2:**
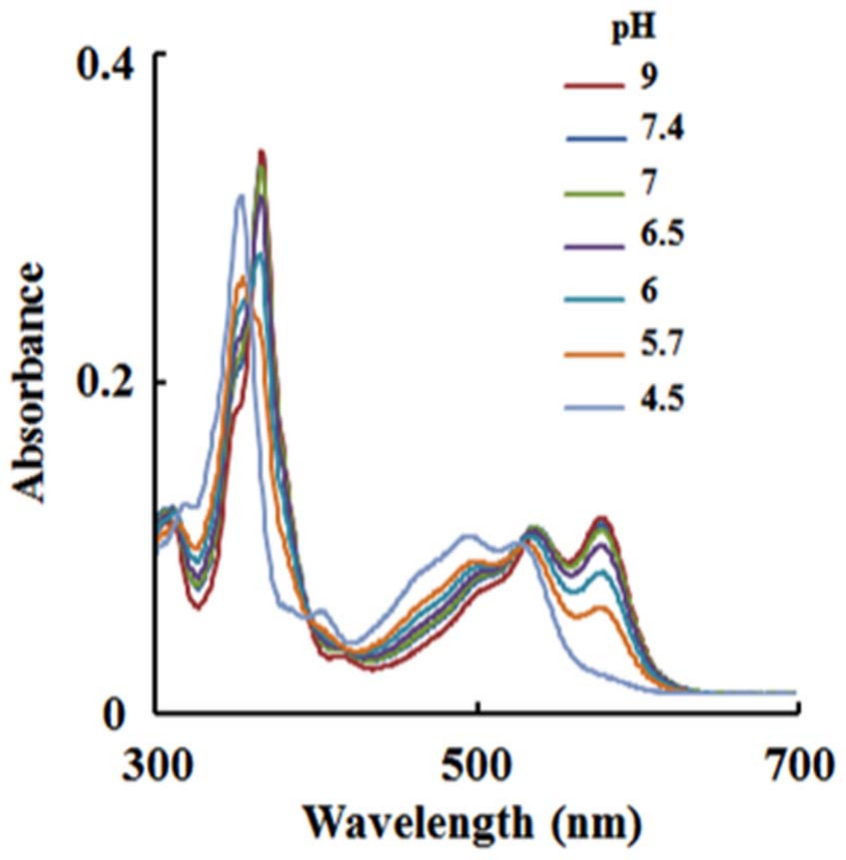
Stability of (CN)_2_-Cbi as a function of pH. UV-visible difference measurements recorded after dissolving equal amounts of (CN)_2_-Cbi (14 µM, final) at different pH values as described under “Materials and Methods.”

We first investigated the stability of (CN)_2_-Cbi as a function of pH. Figure 2 shows the UV-visible absorption spectra of (CN)_2_-Cbi prepared at different pHs ranging from 4.5–9.0. At pH range 6.5–9.0, the spectra exhibited corrin maxima centered at 366 with two resolved visible peaks at 538 and 578 nm. At pH 4.5, the spectra exhibited corrin maxima centered at 354 with visible bands centered at 497, and 526 nm, indicating the replacement of one of the CN^-^ molecules with H_2_O and the formation of an H_2_O-Cbi-CN complex [37]. At pHs between 5.7 and 6 the spectra demonstrated an equilibrium mixture of (CN)_2_-Cbi and (CN)-Cbi-(H_2_O). These results are consistent with those previously reported by Ye et al. in which the instability of Cbi/Cbl at lower pHs was described [38]. The pH profiles of Cl_2_, OCl^-^, and HOCl populations have been previously reported by Kettle et al. [39]. The HOCl population shows a bell-shaped pattern, with optimum pH ranging from 3.0 to 7.0 with a sharp drop to ∼50% at pH 2 (Cl_2_ and HOCl are about equal) and to ∼50% at pH 7.4 (HOCl and OCl^-^ are about equal). At pH greater than 7, OCl^-^ starts to form and becomes the predominant species at pH 9 and above. Collectively, these observations have been used as a guide to investigate the reaction of HOCl against (CN)_2_-Cbi utilizing rapid kinetic measurements.

### Spectrophotometric and rapid kinetic characterization of the interaction between HOCl and (CN)_2_-Cbi

**Figure 3:**
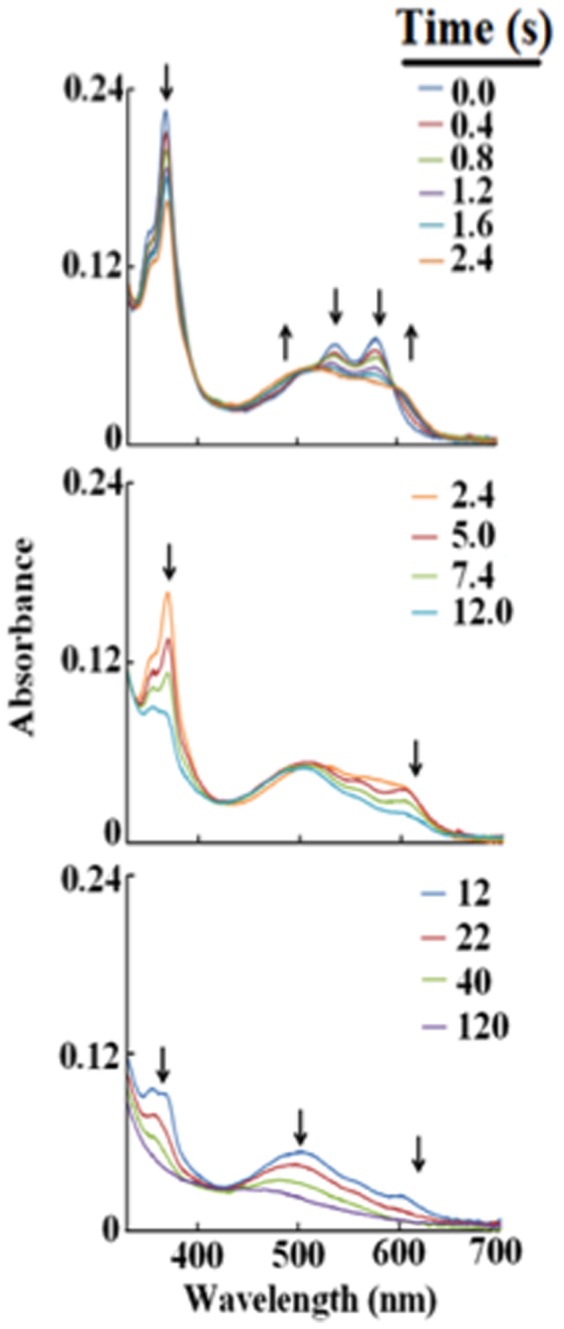
Diode array rapid scanning spectra for the intermediates and corrin ring destruction by reacting (CN)_2_-Cbi with HOCl at three sequential time frames. Panel A, spectra traces collected at 0.0, 0.4, 0.8, 1.2, 1.6, and 2.4 s and was attributed to the replacement of the first molecule of CN^-^ with OCl in (CN)_2_-Cbi. Panel B, spectra traces collected at 2.4, 5.0, 7.4, and 12.0 and were attributed to the replacement of the second molecule of CN^-^ with OCl in (CN)_2_-Cbi. Panel C, spectra collected at 12.0, 22.0, 40.0, and 120.0 s and was attributed to corrin ring destruction. Experiments were carried out by rapid mixing a phosphate buffer solution (200 mM, pH 7.0), at 25°C, supplemented with 20 µM (CN)_2_-Cbi with a same volume of a buffer solution supplemented with 80-fold excess of HOCl. Arrows indicate the direction of spectral change over time as each intermediate advanced to the next. These data are representative of three independent experiments.

We next utilized a diode array stopped-flow spectrophotometer to investigate the kinetics of the reaction of (CN)_2_-Cbi with increasing concentrations of HOCl. Reactions were run in the dark (vitamin B_12_ derivatives have been considered light sensitive analytes) under aerobic conditions, at 25°C, pH 6.4–9.0. At physiologic pH, rapid mixing of a solution of 20 µM (CN)_2_-Cbi with 80-fold molar excess of HOCl resulted in the rapid formation of a transient intermediate that displayed a decrease in the corrin absorbance peak at 366 nm and broad visible bands centered at 493 and 613 nm, typical of a six-coordinate complex Figure 3 (upper Panel). This spectrum differs from that of (CN)_2_-Cbi, whose corrin maxima are centered at 366 with two resolved visible peaks at 538 and 578 nm [37]. This spectrum also differs from that of both α-cyano, β-aqua-cobinamide (UV/Vis 353, 496, and 525 nm) and α-aqua, β-cyano-cobinamide (UV/Vis 354, 497, and 527 nm) [37]. The spectrum for (CN)_2_-Cbi reported by Zhou and Zelder that was also supported by ^1^H NMR [37] matches our spectrum indicating that (CN)_2_-Cbi is the starting compound. Furthermore, there is no alteration in the (CN)_2_-Cbi upon the addition of excess CN^-^ to the reaction mixture solidifying the evidence in favor of (CN)_2_-Cbi as the starting compound. The spectrum of the intermediate that initially formed following addition of HOCl/OCl^-^ to (CN)_2_-Cbi is consistent with the replacement of one of the CN^-^ groups with hypochlorite ion (OCl^-^). This Cbi intermediate formed within 2.4 s after mixing, but was unstable and rapidly converted into a more stable intermediate within 12 s, as judged by further decrease in the absorbance at 366 nm and a time-dependent shift in visible absorbance peak from 613 to 493 nm. These spectral changes were attributed to the replacement of the second CN^-^ group with OCl^-^, as shown in Figure 3 (middle Panel). The second transient intermediate that formed within 12 s of initiating the reaction was also unstable and this was followed by spontaneous corrin ring destruction, within minutes of initiating the reaction (Figure 3, lower Panel). This conclusion was made by the decrease and flattening in the absorbance spectra indicating oxidative destruction of the corrin ring. Spectral transitions between each intermediate formed revealed distinct and well-defined isosbestic points (Figure 3). Thus sequential formation and decay of Cbi intermediates occur at sufficiently different rates to enable each process to be studied by conventional (i.e., single mixing) stopped-flow methods. Similar intermediates were observed when the experiments were repeated at pH 6.4 and 9.0 (where HOCl and OCl^-^, respectively, predominated); however the rate constants of the transition from one intermediate to another were slower (see below). Collectively, these results indicate that HOCl binds (CN)_2_-Cbi generating two unstable transient intermediates before corrin ring fragmentation. The generation of the transient octahedral intermediate, (OCl)(CN)-Cbi, and its conversion to (OCl)_2_-Cbi was characterized by one set of isosbestic points at 520 and 602 nm, as well as differing responses to varying HOCl concentrations.

**Figure 4:**
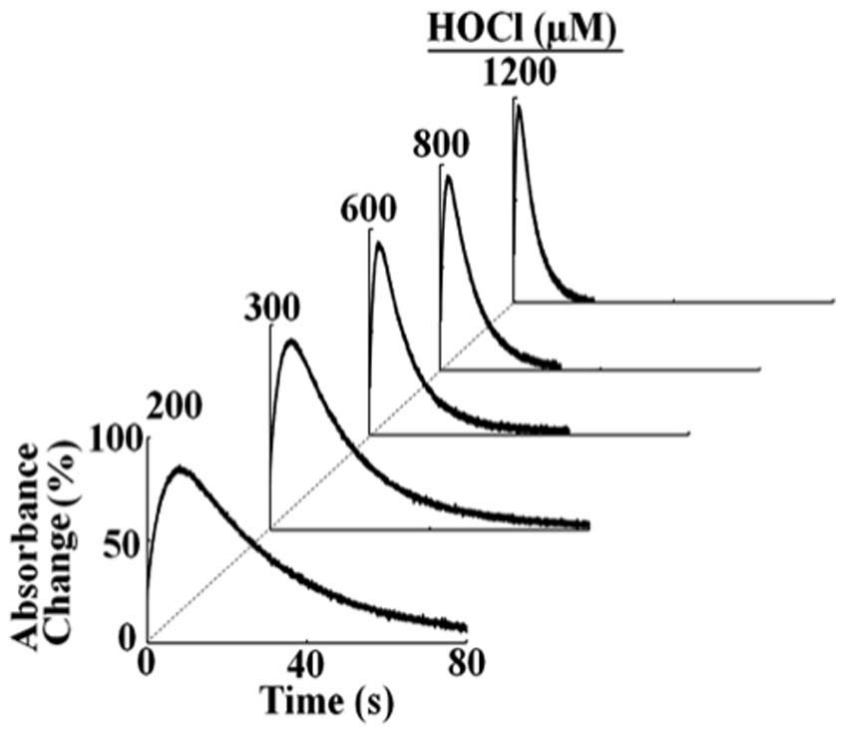
The effect of HOCl concentration on the formation, duration of (OCl) (CN)-Cbi and its conversion to (OCl)_2_-Cbi. A solution containing sodium phosphate buffer (200 mM, pH 7.0) supplemented with 5 µm (final) dicyanocobiamide was rapidly mixed with an equal volume of buffer containing increasing concentrations of HOCl (200, 300, 600, 800, and 1200 µM, final) at 25°C. Replacement of the first CN^-^ molecule by OCl^-^, duration, and its decay to (OCl)_2_-Cbl were monitored as a function of time by observing spectral changes at 613 nm. The final concentration of HOCl in mixtures is indicated.

**Figure 5:**
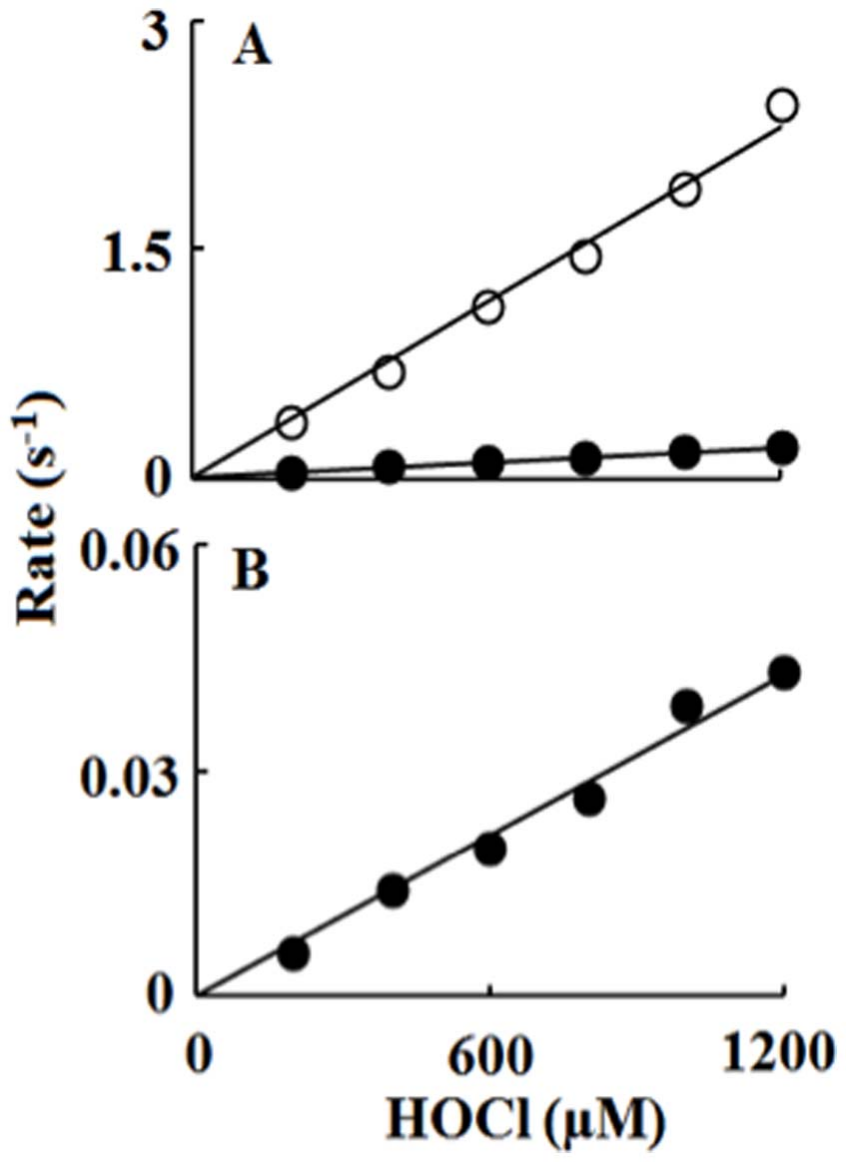
Rate constants of the axial ligands replacement and corrin ring destruction of dicyanocobinamide as a function of HOCl concentration. Upper panel, the observed rate constants of (OCl)(CN)-Cbi complex formation (open circles) and its conversion to (OCl)_2_-Cbi (monitored at 613 nm) (closed circles) observed in Fig. 3 were plotted as a function of HOCl concentration. R^2^ values for the first and second phases were 0.992 and 0.995 respectively. Data represent the mean of triplicate determinations from an experiment performed three times. Lower panel, the rate constants for the corrin ring destruction, for the same reaction, monitored at 493 nm as a function of HOCl concentration. R^2^ value was found to be 0.987. These data are representative of three independent experiments and the standard error for each individual rate constant has been estimated to be less than 8%.

We next utilized single wavelength stopped-flow to elucidate the mechanism by which HOCl mediates ligand replacement and corrin ring destruction. At pH 7.4 when the reaction was monitored at 613 nm, there was a rapid increase in absorbance which reached a maximum intensity in less than 10 s, and then decayed over a period of approximately 100 s when a solution of 10 µM (CN)_2_-Cbi was rapidly mixed with 200 µM HOCl (Figure 4). The time course for this reaction was fitted to Eq. 2 with values of k_1_ and k_2_ of 0.30 and 0.026 s^−1^, respectively. The increase in absorbance at the start of the reaction can be attributed to axial ligand replacement of one of the CN^-^ groups while the subsequent decrease in absorbance that took place over the next 100 s can be attributed to the replacement of the second CN^-^ group. These experiments were conducted with fixed amounts of (CN)_2_-Cbi with increasing concentrations of HOCl. The addition of increasing concentrations of HOCl to (CN)_2_-Cbi resulted in dramatic effects on the rates of the buildup, duration, and decay as assessed by stopped-flow spectroscopy (Figure 4). HOCl was readily used as a ligand of (CN)_2_-Cbi, as indicated by the enhanced rate of this intermediate formation (Figures 3 and 4). As shown in Figure 5A, the rate of both phases increased linearly when plotted as a function of HOCl concentration, yielding second-order rate constants of 0.002 µM^−1^ s^−1^ and 0.0002 µM^−1^ s^−1^ for the first and second phases, respectively. Figure 3 shows the decrease in absorbance at 613 nm that took place during the next 100 s of the reaction when increasing concentrations of HOCl were rapidly mixed with a fixed concentration of (CN)_2_-Cbi (10 µM). This decrease and flattening in the spectra associated with a lack of isosbestic points was attributed to Cbi destruction that could be due to the loss of hyperconjugation in the molecule, Co atom release, and/or Cbi fragmentation. Plots of HOCl concentration versus observed rates of corrin destruction demonstrated linear kinetics and yielded a second-order rate constant (k_3_) of 4.0×10^−5^ µM^−1^ s^−1^ (Figure 5B). In all cases, the plots intersected the axes near the origin indicating that the transient intermediates formed and subsequent Cbi corrin destruction are essentially irreversible (Figure 5).

At pH 6.5 where HOCl is the predominant species in the solution there is little or no effect on the rate constants or the intermediates that formed upon mixing HOCl with (CN)_2_-Cbi compared to pH 7.4, as judged by single wavelength stopped-flow measurements (Figure 7). Rapid kinetic measurements performed at pH 9, where OCl^-^ is the predominant species in the solution, revealed that the rate constants of ligand replacement and corrin ring destruction were 3–4 fold lower than those observed at a lower pH range (6.5–7.4) (Figure 7), indicating the stability of (CN)_2_-Cbi at this pH range.

Corrin ring destruction can also occur at a lower pH (4–5) where one of the CN^-^ molecules is replaced with H_2_O (data not shown). Importantly, replacement of one of the CN^-^ molecules with H_2_O did not show any sign of protection against HOCl destruction. The characteristic spectrum of (CN)-Cbi-(H_2_O) was also observed when (CN)_2_-Cbi was mixed with low concentration of HOCl (eg., 1∶1, or 1∶2 mole equivalent of (CN)_2_Cbi∶HOCl, respectively). The (CN)-Cbi-(H_2_O) complexes that formed at low HOCl concentrations were identified by liquid chromatography mass spectrometry (LC-MS) analysis and rapid kinetic measurements (data not shown).

In increase in the temperature from 25 to 37°C nearly doubles the rate of the reactions, but the kinetic behavior and the sequence of the reaction stays the same. We selected to show the results at 25°C to slow the reaction for clarity purposes.

### Liberation of CNCl and Co from (CN)_2_-Cbi after HOCl treatment

**Figure 6:**
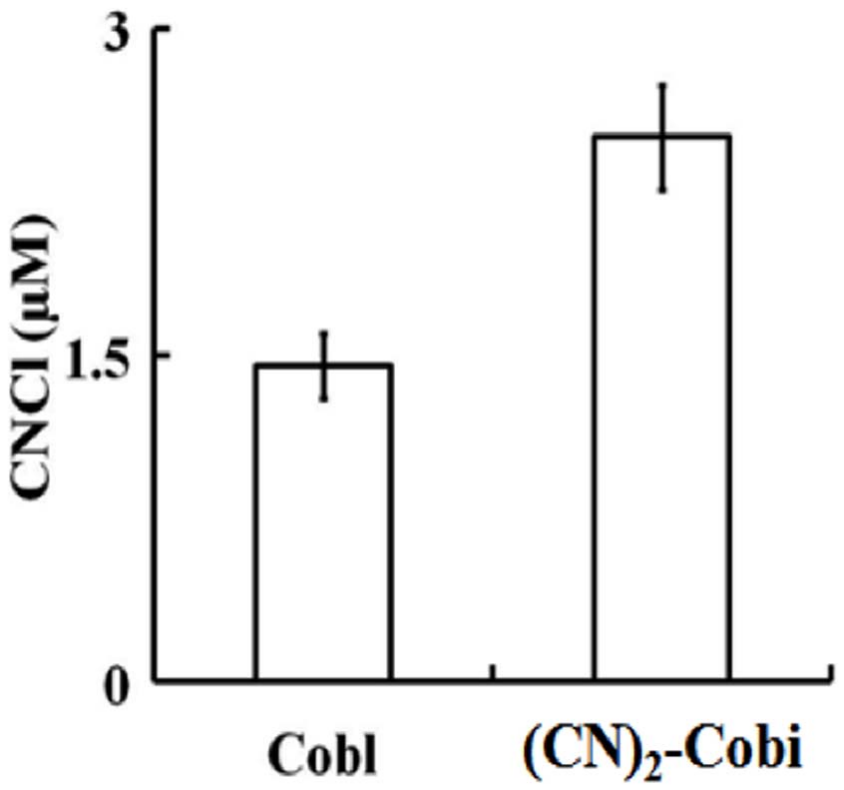
Cyanocobalamin and (CN)_2_-Cbi destruction mediated by HOCl causes the liberation of CNCl. Equal concentrations of Cbl and (CN)_2_-Cbi (110 µM) were treated with 50-fold molar excess of HOCl and CNCl generation were assayed colorimetrically as detailed under Materials and methods. The data are representative of three independent experiments with the error bars representing the standard error measurements.

We next investigated whether the CN^-^ released from the (CN)_2_-Cbi molecule reacts with the excess HOCl in the reaction mixture to form cyanogen chloride (CNCl). Dicyanocobinamide (110 µM) was treated with 50-fold molar excess of HOCl and the accumulation of CNCl was measured using the pyridine-1,3 dimethyl barbituric acid colorimetric assay as previously reported. As shown in Figure 6, the amount of CNCl generated from the reaction of (CN)_2_-Cbi with HOCl was approximately 1.8 fold the amount generated from the same reaction of Cbl with HOCl. Thus, the destruction of (CN)_2_-Cbi mediated by HOCl is more toxic than that of Cbl.

**Figure 7:**
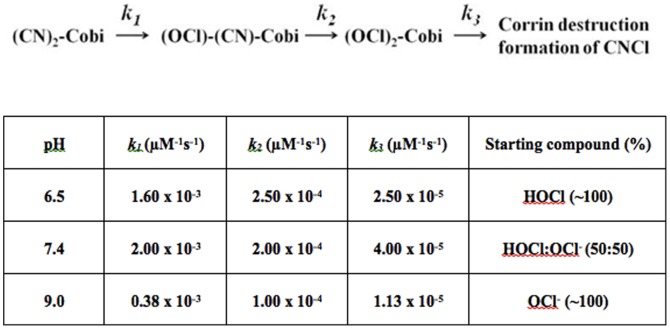
Rate constants for the formation and decay of the intermediates that formed upon mixing (CN)_2_-Cbi with HOCl. (CN)_2_-Cbi was rapid-mixed with buffer containing HOCl at various concentrations. Rates of complex formation and decay were determined at three different pHs (6.5, 7.4, and 9.0) by following absorbance change at 613 or 493 nm, at 10°C, and the rate constants determined as described in the text. These data are representative of three independent experiments and the standard error for each individual rate constant has been estimated to be less than 3%.

In contrast to what was observed for the reaction of hemoproteins and free heme with HOCl where we detected Fe^2+^ release [40]–[43], we were unable to determine the oxidation state of the Co ion and the nature of the axial ligation to the Co after corrin ring destruction by HOCl. This is due to several different smaller fragments of the corrin ring generated that might coordinate to Co. Colorless Cobalt (II)/(III) complexes have previously reported [44]. The exact nature of the Co-complex is currently under investigation in our laboratory. Collectively, HOCl not only mediated (CN)_2_-Cbi ring destruction, but also mediated the generation of CN-/CNCl.

## Discussion

In this work, we showed that HOCl mediates (CN)_2_-Cbi corrin ring destruction and subsequent liberation of toxic CNCl. Corrin ring destruction occurs through a mechanism that initially involved the sequential replacement of the CN^-^ groups by OCl^-^ molecules. Changes in the corrin ring geometry either from the alteration in oxidation state of the Co atom or by the replacement of the axial ligands might make the ring more susceptible to HOCl-mediated oxidation and destruction. Thus, secondary to the liberation of toxic CNCl/HCN/CN^-^ molecules, cyanocobalamin derivatives may display harmful effects in chronic inflammatory states where HOCl is elevated.

In general, the characteristic spectroscopic features of vitamin B_12_ derivatives are extremely sensitive to the alteration of both the axial ligands, the modification of the corrin ring, as well as the oxidation state of the Co atom [45], [46]. These events may have various effects on the geometric parameters of Cbi including the modulation of the Co atom's electronically charged microenvironment by changing a variety of factors. These include the location of the Co atom (in the plane or out of the plane of the corrin ring), the distance between the Co atom and the axial ligands, the distance between the Co atom and the four Ns of the pyrrole rings, the corrin ring fold angle, and the Co-ligand bond angles and their ability to make Van der Waals contact with the corrin ring [47]. There is a considerable amount of evidence from several X-ray crystallographic studies that indicate that these parameters are interconnected and alteration of one modulates the others [13]. In (CN)_2_-Cbi, the coordination around the central Co atom is a distorted octahedron [47]. Although the Co-C and C-N bond lengths of both axial ligands along with the Co-C-CN angles are almost equal (within the experimental error), the X-ray analysis indicated that the two CN^-^ molecules located at both axial ligands exhibit differences in conformation [47]. These differences resulted, namely, from the orientation of the two CN^-^ molecules in two different directions. Indeed, one of the N-C-Co linkages is consistently bent away from the bond linking the two-pyrrole groups directly while the other linkage can take up a variety of orientations. This flexibility allows for the interaction of the corrin edge to adjacent atoms of the side chains with CN^-^, and the occurrence and effects of specific CN-H bonding [47]. These events, accompanied by changes in the corrin ring fold angle, may cause a steric effect that masks one site and minimally restricts access of the ligand replacement to the other site. This behavior is an exceptional case among other tetrapyrrole compounds and explains why the replacement of CN^-^ molecules at both axial sites happened sequentially rather than simultaneously when a fixed amount of (CN)_2_-Cbi was rapidly mixed with increasing concentrations of HOCl.

The shift and alteration in the UV-visible spectra indicate that the ligand replacement of the first CN^-^ molecule by OCl^-^ and the formation of the (OCl)(CN)-Cbl complex displays distinct effects on the corrin Co microenvironment. This is expected, since the OCl^-^ molecule has different physical and chemical properties such as ion size, electronegativity, and affinity toward Co. Given the radius, polarizability, flexibility, and charge of OCl^-^ compared to CN^-^, OCl^-^ displays the potential capacity to adopt a bent geometry and bind (CN)_2_-Cbi to form a six-coordinate complex. The interactions of (OCl)(CN)-Cbi with the second OCl^-^ molecule suggest that the bond of OCl^-^ has electronic influences on the Co-bound CN. Hypochlorite, like other bulky ligands, may display a longer cobalt-ligand (Co-L) bond and a large corrin ring fold angle due to a large area of contact (Van der Waals) with the corrin ring which shortens the trans Co-L bond [48]. Therefore, replacement of the first CN^-^ with OCl^-^ in (CN)_2_-Cbi is likely accompanied by a strengthening of the remaining Co-CN linkage which may allow the Co ion to move away from the corrin ring center and closer to the CN^-^ molecule. Such a process may increase the affinity of the corrin ring towards CN^-^, which ceases the pyrrole ring movement. Thus, replacement of the second CN^-^ molecule with OCl^-^ should be more difficult than that observed for the first CN^-^ molecule. This notion is supported by the slower rate constant of OCl^-^ binding to the second site.

The perturbation in the electronic structure of the corrin ring mediated by HOCl modification and oxidation may also play an important role in deactivating the axial coordination site toward ligand substitution. This assumption is built on a previous study by Brown et al., which showed that the substitution of the C10 by an electron-withdrawing group such as NO deactivates the axial coordination site toward ligand substitution [48]. Chemaly et al., have also demonstrated that modifications of C10 and C5, in which the H atoms are replaced by Cl and OH, respectively, depend strongly on the polarizability of the axial ligand [49]. The hypochlorite ion is a more stable and less active form of chlorine. It is still capable of replacing both CN^-^ molecules of (CN)_2_-Cbi and mediating corrin ring destruction but at slower rates. Indeed, our rapid kinetic measurements reveal that the rate constants of ligand replacement and corrin ring destruction are 3–4 fold lower than those observed at a lower pH where HOCl is the predominant species (Figure 7). A mechanism that describes the involvement of HOCl in tetrapyrrole macrocyclic compound destruction (e.g. corrin ring, heme, and porphyrin) that could apply to (CN)_2_-Cbi corrin ring damage was described by Maitra et al., and Abu-Soud et al., [8], [40]. In this model, we showed that following ligand replacement, HOCl oxidatively cleaved the corrin ring leading to the generation of a dicorrinic derivative. The attack of HOCl to the carbon–methyne bridges between the adjacent corrin rings led to the formation of chlorinated adducts, which by releasing chloride, formed an epoxide or aminal. The epoxide gave rise to a hydroxylated compound with the hydroxyl group being attached to the carbon-methyne bridge of the tetrapyrrole moiety, where the initial attack by HOCl occurred.

Comparison of the kinetic data obtained for the reaction of (CN)_2_-Cbi with HOCl showed in Figures 3–5 with recently reported reactions of Cbl with HOCl showed the involvement of several kinetically and spectrophotometrically distinguishable marks. Interaction of HOCl with Cbl consists of a three-step mechanism: a lag phase interval parallel to the weakening and release of the α-axial ligand; α-axial ligand replacement leading to the formation of a new hexa-coordinated intermediate; and corrin destruction with subsequent CNCl formation [8]. The dependence of the pseudo-first-order rate constants of the two initial phases on the concentration of HOCl are noticeably curved indicating that these reactions are second order with respect to HOCl and follow the rate expression (k_obs_ = k_1_[HOCl]^2^) with second order rate constants for the first and second phases of 1×10^−7^ µM^−2^s^−1^ and 3×10^−7^ µM^−2^s^−1^, respectively [8]. It was concluded that the chlorinated intermediate was generated through two consecutive steps based on the similar trends and close proximity of the values of the rate constants for the first and second steps. The rate constant of Cbl destruction is relatively slow with a second order rate constant of (2×10^−5^ µM^−1^s^−1^). The second order rate constant of HOCl mediated Cbl corrin ring destruction is comparable to the rate constants of HOCl with (CN)_2_-Cbi and other biomolecules such as C = C in lipid molecules, adenine mononucleotide phosphate, and backbone amides in protein chains (for a detailed review see [23], [50]). Multiple fragments were identified utilizing HPLC and mass spectrometry when Cbl was treated with HOCl in phosphate buffer associated with a buildup of CNCl. Similar products have been obtained when the reaction was carried out in plasma, indicating the biological relevance of our finding [40]. A biphasic reaction has also been reported for CN^-^ with (OH)(H_2_O)-Cbi to form (CN)_2_-Cbi [2], [45]. In this reaction, the replacement of the H_2_O molecule by CN^-^ has been suggested as an initial step due to favorable thermodynamic and kinetic reasons, subsequently (OH)(CN)-Cbi was attacked by the second CN**^-^** molecule to form (CN)_2_-Cbi. In addition, Baldwin et al. [51] have reported that cyanoaquacobinamide reacts with CN^-^ much faster than either (H_2_O)_2_-Cbi or (H_2_O)(OH)-Cbi due presumably to the stronger *trans*-labializing effect of CN^-^ compared to OH^−^ [2], [45].

Sharina et al., showed that (CN)_2_-Cbi acts as a sGC coactivator both in vitro and in intact cells [19]. Heme depletion or heme oxidation does not affect (CN)_2_-Cbi-dependent activation. In addition to their antimalarial activity, a recent study by Weinberg et al. has shown that cobalamins and cobinamides inhibit the three NOS isoforms to various degrees [21]. The authors suggested that these compounds could be administered acutely, subacutely, or chronically in certain diseases in which NO acts in a deleterious fashion (e.g., inflammatory diseases) with very few side effects. Our current finding opposes this implication. While cobalamins are widely used to treat human cyanide poisoning because of their efficacy and high affinity for CN^-^, exposure of (CN)_2_-Cbi to HOCl can undermine these beneficial effects and generate proinflammatory reaction products, such as CNCl. Hypochlorous acid mediates corrin ring destruction and liberates toxic molecular products such as CN^-^/HCN/CNCl, all potent blood agents. More recently, we have shown that HOCl can promote heme destruction in free heme, hemoproteins, and red blood cells leading to iron release and protein aggregation [40]. Our work, thus, provides some exciting evidence to support the potential relationship between elevated levels of free metals and elevated activity of MPO. This deleterious relationship, during inflammation, could manifest in lipoprotein oxidation in vivo, the initiation of immunologic reactions, and the production of damaging ROS such as hydroxyl radicals (•OH) via a Fenton-like reaction when reducing agents are available (Cl^-^ can reduce Co^3+^ to Co^2+^ [33], [52]. The higher toxicity of (CN)_2_-Cbi destruction mediated by HOCl compared to Cbl destruction is reflected by the liberation of almost twice the amount of CNCl. The overall kinetic mechanism that describes our current finding is shown in Figure 7. Thus, supplementation with HOCl scavenger may provide a beneficial effect through prevention of the formation of CNCl. Indeed, previous studies by Matthews et al. have shown that treatment of HL60 cells (cells expressing MPO) by Cbl led to cytotoxicity, which could be prevented by adding methionine, a potent scavenger of HOCl, to the medium [53].
